# Detection of West Nile virus in a common whitethroat (*Curruca communis*) and *Culex* mosquitoes in the Netherlands, 2020

**DOI:** 10.2807/1560-7917.ES.2020.25.40.2001704

**Published:** 2020-10-08

**Authors:** Reina S Sikkema, Maarten Schrama, Tijs van den Berg, Jolien Morren, Emmanuelle Munger, Louie Krol, Jordy G van der Beek, Rody Blom, Irina Chestakova, Anne van der Linden, Marjan Boter, Tjomme van Mastrigt, Richard Molenkamp, Constantianus JM Koenraadt, Judith MA van der Brand, Bas B Oude Munnink, Marion PG Koopmans, Henk van der Jeugd

**Affiliations:** 1 Viroscience, ErasmusMC, Rotterdam, the Netherlands; 2 Institute of Environmental Sciences, Leiden University, Leiden, the Netherlands; 3 Vogeltrekstation —Dutch Centre for Avian Migration and Demography, NIOO-KNAW, Wageningen, the Netherlands; 4 Naturalis Biodiversity Center, Leiden, the Netherlands; 5 Division of Pathology, Utrecht University, Utrecht, the Netherlands; 6 Dutch Wildlife Health Center (DWHC), Utrecht, the Netherlands; 7 Laboratory of Entomology, Wageningen University and Research, Wageningen, the Netherlands; 8 Wildlife Ecology and Conservation group, Wageningen University and Research, Wageningen, the Netherlands; 9 Department of Animal Ecology, Netherlands Institute of Ecology (NIOO-KNAW), Wageningen, the Netherlands

**Keywords:** West Nile Virus, Europe, the Netherlands, zoonotic infections, viral infections, West Nile fever, outbreaks, surveillance, epidemiology, molecular methods, sequencing

## Abstract

On 22 August, a common whitethroat in the Netherlands tested positive for West Nile virus lineage 2. The same bird had tested negative in spring. Subsequent testing of *Culex* mosquitoes collected in August and early September in the same location generated two of 44 positive mosquito pools, providing first evidence for enzootic transmission in the Netherlands. Sequences generated from the positive mosquito pools clustered with sequences that originate from Germany, Austria and the Czech Republic.

Extensive surveillance of mosquitoes, dead and live birds was set up in 2016 to monitor the introduction and spread of a selection of high-risk arboviruses in the Netherlands. The presence of Usutu virus (USUV) infections was confirmed first in 2016, and genomic sequencing provided evidence for continued enzootic presence of USUV in subsequent years [1,2]. West Nile virus (WNV), however, had so far not been detected. Here we report the first locally acquired WNV detection in birds in the Netherlands.

## Surveillance in mosquitoes, dead birds and live birds

**Figure 1:**
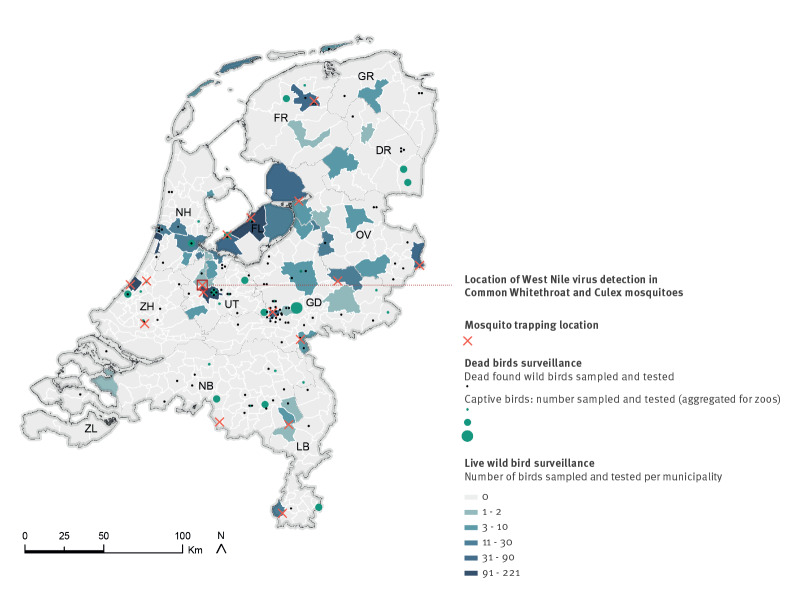
Surveillance network for zoonotic viruses in birds and mosquitoes and location of West Nile Virus detection, the Netherlands, 2020

DR: Drenthe; FL: Flevoland; FR: Friesland; GD: Gelderland; GR: Groningen; LB: Limburg; NB: Noord Brabant; NH: Noord Holland; OV: Overijssel; UT: Utrecht; ZH: Zuid Holland; ZL: Zeeland.

**Figure 2:**
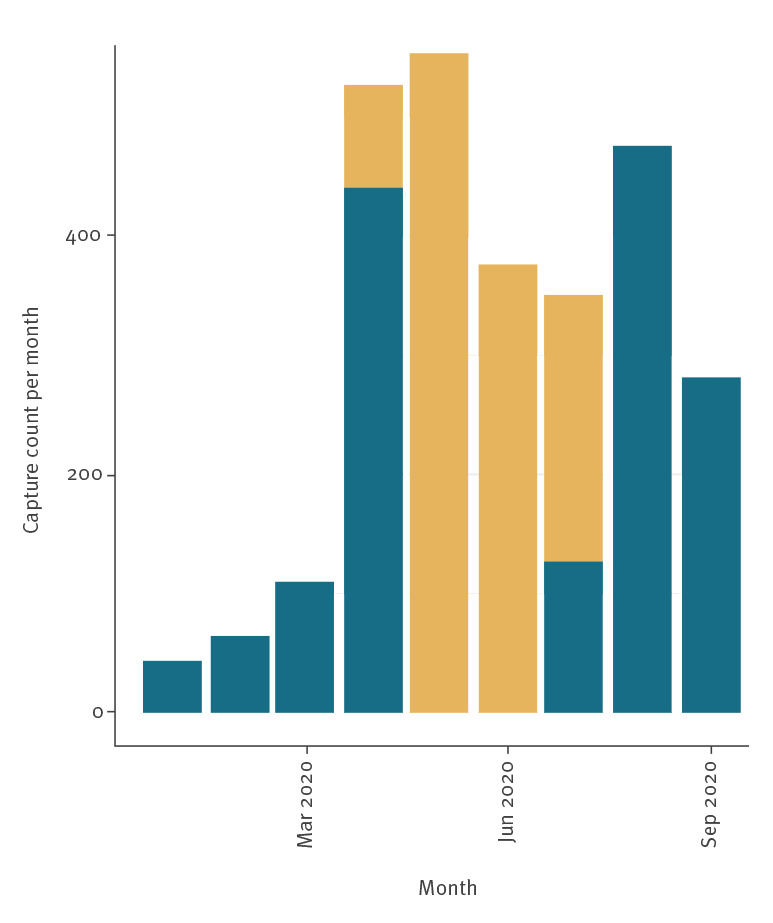
Number of live birds caught and sampled, the Netherlands, 2020 (n = 2,783)

Since January 2020, 2,783 live birds have been caught and sampled in the Netherlands to detect possible introduction and spread of zoonotic viruses. Wild birds were randomly captured using mist nets and other trapping methods and sampled as part of a wider study of the presence of zoonotic viruses in birds in the Netherlands [1,2]. Captured birds were ringed, weighed and measured, sex and age were determined, and throat (and for larger species cloacal) swabs were collected and stored in virus transport medium at −80 °C until use. Recapturing and resampling of ringed birds occurs frequently during the breeding season. Because of COVID-19, it was not possible to test samples received between 29 April and 24 July. To date 1,477 of 2,783 birds have been tested for WNV (Figures 1 and 2).

Crosses indicate mosquito trapping locations. Dots indicate locations of dead birds collected in 2020. Captive dead birds are aggregated by zoo. The number of live birds sampled and tested in 2020 is indicated at the municipality level. Source administrative boundaries: Statistics the Netherlands (CBS). Created using ArcGIS 10.6 software by ESRI.

Yellow: samples that have been sampled, but not yet tested owing to COVID-19. Blue: samples that have been tested for West Nile virus.

Mortality in captive and wild birds is reported through a citizen science-based alerting system. In 2020, a total of 221 birds were reported and collected for further research (Figure 1). For all birds, an autopsy was performed and brain samples were taken.

In parallel to the sampling of live and dead birds, a network of 16 mosquito trapping sites was set up in July 2020. At each site, mosquitoes were collected on a weekly basis using molasses fermentation-baited BG-pro traps (Biogents GmbH, Regensburg, Germany) without additional lures, following a slightly modified molasses fermentation protocol [3]. The mosquitoes were morphologically identified. Individuals belonging to the *Culex pipiens* complex were pooled, based on trap location and time point of sampling, with a maximum of 10 individuals per pool. Mosquito pools were homogenised in medium (DMEM, NaHCO_3_, Hepes-buffered saline, penicillin/streptomycin, amphotericin).

All samples (brain samples of dead birds, throat and cloaca (pooled, per bird), swabs of live birds and mosquito pools) were screened for the presence of WNV and USUV using a real-time PCR with primers and probes as described previously, with phocine distemper virus (PDV) used as an internal control [4]. Positive results were confirmed with a second PCR targeting another region of the genome (Table). In addition, all RT-PCR-positive samples were subjected to sequencing.

**Table:** Primer sequences for screening and confirmation RT-PCR for West Nile virus, the Netherlands, 2020

Virus	Forward primer '5 -> 3'	Reverse primer '5 -> 3'	Probe '5 -> 3'	Goal
WNV	CCACCGGAAGTTGAGTAGACG	TTTGGTCACCCAGTCCTCCT	TGCTGCTGCCTGCGGCTCAACCC	Screening
WNV lineage 2	CCATCTGYTCCGCWGTGCC	ATCCATTCTCCTTTTGCGTGRAT	TGGGTTCCCACRGGGCGYACCACYTG	Confirmation

WNV: West Nile virus.

## Results of the West Nile virus screening

One bird, a common whitethroat (*Curruca communis*) was caught on 22 August in the municipality of Utrecht, the Netherlands and tested positive for WNV lineage 2 (cycle threshold (Ct) value: 31.8 and 32.5 in the screening and confirmation PCR, respectively). The bird appeared healthy at the time of capture. The USUV RT-PCR was negative. The bird was caught multiple times at the same location between 2018 and 2020. On 21 July 2018 the bird was in first year plumage. In May 2020, the bird was identified as a sexually active adult male based on the shape of its cloaca and tested negative for WNV. A total of 173 birds caught at the same location in 2020 tested negative for WNV.

At the same location, two of 44 pools of *C. pipiens* mosquitoes tested positive for WNV RNA. Positive pools were collected in August and September 2020, and came from two traps ca 250 m apart. The first pool (Ct value: 18.6) was collected in the same week as the positive bird (17–23 August). The second positive pool (Ct value: 26.1) was collected in the week from 31 August to 6 September. Mosquitoes from other locations have not yet been screened for WNV.

## Sequencing

**Figure 3:**
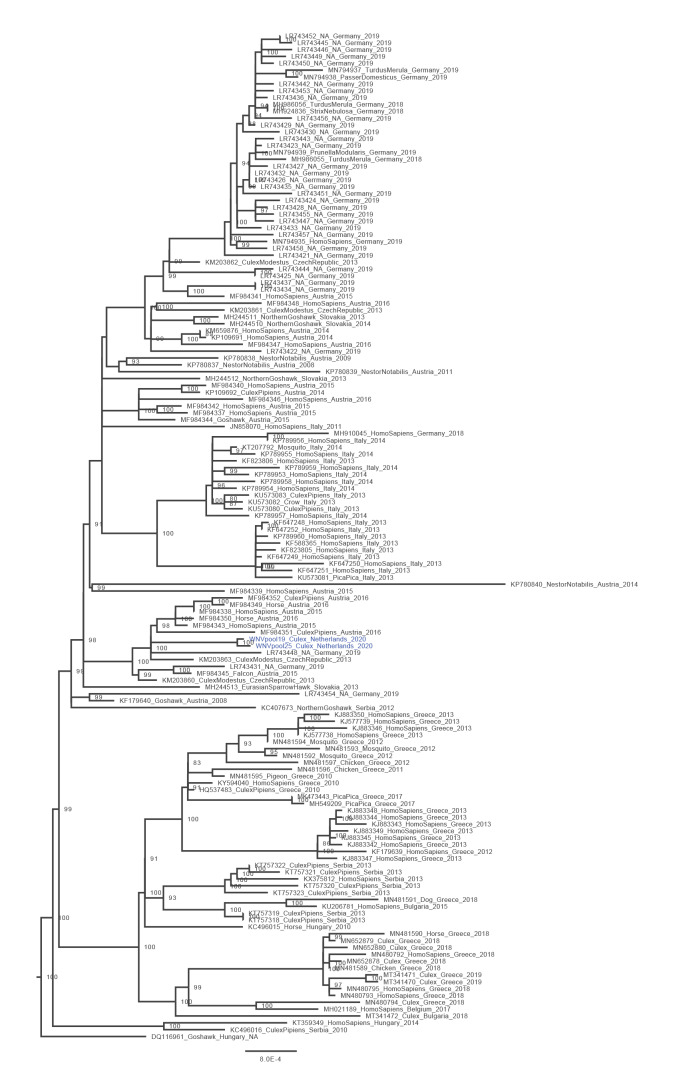
Phylogenetic tree of all full genomes of West Nile virus lineage 2 available in GenBank on 4 September 2020 (n = 147)

We developed a WNV-specific amplicon-based whole genome sequencing PCR similar to what has previously been described for USUV and SARS-CoV-2, using Nanopore sequencing [2,5]. Sequencing was performed as previously described [2,5]. Primer sequences are available in Supplementary Table S1. We downloaded all full-length WNV sequences available in GenBank on 4 September 2020 [6] and aligned them using MUSCLE software [7]. We downsampled the number of reference sequences to include all lineage 2 WNV sequences, but only a small selection of WNV lineage 1. Phylogenetic analysis using IQ-TREE [8] under the GTR + F + I + G4 model, as determined by the best model prediction option, confirmed the presence of WNV lineage 2 in the two mosquito pools for which we had nearly complete genome coverage (Figure 3). The viral load in the bird sample was too low for full genome sequencing but yielded a 500 bp sequence matching with lineage 2 WNV. The genomes from the two mosquito pools (Genbank accession numbers MW036633; MW036634) differed by 3 nt from each other and clustered with sequences that originate from Germany (in 2019) and older sequences found in Austria and the Czech Republic (Figure 3).

The figure includes two sequences acquired from two mosquito pools in this study, in blue. The scale bar represents units of substitutions per site.

## Discussion

Most human and animal cases of WNV in Europe are reported from countries in the southern and south-eastern parts but the geographic distribution has expanded considerably over the past 10 years, from six countries reporting human or animal cases in 2010 in the European Union (EU) to 12 in 2019 [9,10]. In 2020, a total of 209 human cases have been reported so far in the EU, in Greece, Italy, Romania, Hungary and Spain, as well as multiple asymptomatic cases found in blood donor screening and nine clinical cases in humans in Germany [11]. Considering that most people do not develop symptoms and only human cases with known place of infection on NUTS3 level are included in the overviews from the European Centre for Disease Prevention and Control (ECDC), these numbers are likely to be an underestimation.

In contrast to the United States, the main amplification host species for WNV in Europe have not been defined with certainty [12]. However, a wide range of birds is susceptible, including a variety of migrating bird species, which can play an important role in the national and international spread of the virus [13]. Mosquitoes can acquire the virus by feeding on a viraemic bird and can transmit it to other birds, animals or humans following an intrinsic incubation period [14]. The main vectors in Europe belong to the *C. pipiens* complex, which are present throughout Europe [15]. Moreover, northern and north-western *Culex* mosquitoes have been shown to be highly competent for both WNV lineages, and wild-caught Dutch jackdaws and carrion crows are susceptible experimentally [15-18]. Humans and equids are considered dead-end hosts but in highly enzootic areas, a much broader range of species can be infected [19]. During the 2020 transmission season, WNV infections in equids were observed in France, Spain, Italy, Germany and Portugal but only in Germany, WNV circulation in birds was officially reported. In contrast to humans and equids, reporting of WNV in birds is not notifiable [11]. 

The simultaneous detection of WNV in a local common whitethroat and *Culex* mosquitoes provides definitive evidence for enzootic transmission in the Netherlands. In 2016, five of 265 screened bird serum samples collected in the Netherlands were found WNV antibody-positive [20], but no virus was found. The two nearly complete whole genome sequences differed from each other by 3 nt, which could indicate local evolution, although separate introductions are also possible. The most recent sequences that clustered with the Dutch WNV sequences originated from Germany, which may be the origin of the virus we found.

Common whitethroats are long-distance migrants that arrive in the Netherlands in the second half of April, while passage of more northern populations occurs until the end of May. Post-breeding dispersal is notable from the beginning of July and southward migration starts in August. Extensive ringing data indicate that nearly all whitethroats captured in the Netherlands between April and July have a local origin [21]. Of the six previous local captures of the positive bird, two occurred in the breeding season of a sexually active male, indicating that this bird was a local breeder. In addition to the negative WNV result in May 2020 and the finding of the WNV-positive mosquitoes at the same site, this strongly suggests that the bird has contracted the virus locally.

The detection in the bird and two positive mosquito pools followed a heatwave, lasting 13 days, including 8 days with temperatures above 30 °C, and average minimum temperatures just below 20 °C [22]. Elevated temperatures are known to shorten the viral extrinsic incubation period in mosquitoes, thereby increasing transmission efficiency. Moreover, virus replication increases, further facilitating arbovirus circulation. 

This finding was the result of an extensive surveillance programme in live birds, dead birds and mosquitoes. To date, no locally acquired human or equine cases have been reported in the Netherlands, but it has been shown that detection of WNV circulation in domestic birds or mosquitoes can precede the first human detections by several weeks [23]. Therefore, we will increase our surveillance effort in the area where WNV was first detected as well as areas that are most at risk of WNV establishment according to a previous risk assessment in the Netherlands [24]. Increased monitoring will encompass surveillance in live and dead birds as well as humans, mosquitoes, livestock, equids and wildlife using a multidisciplinary One Health approach.

## Supplementary Data

157000 Supplement
