# Author’s correction for Euro Surveill. 2020;25(40)

**DOI:** 10.2807/1560-7917.ES.2020.25.43.201029c

**Published:** 2020-10-29

**Authors:** Eurosurveillance editorial team

**Affiliations:** 1 European Centre for Disease Prevention and Control (ECDC), Stockholm, Sweden

In the article “Detection of West Nile virus in a common whitethroat (*Curruca communis*) and *Culex* mosquitoes in the Netherlands, 2020" by Sikkema et al, published on 8 October 2020, corrections were made on request of the authors: The sentence “Mosquitoes can acquire the virus by feeding on a viraemic bird and can transmit it to other birds, animals or humans following an *intrinsic* incubation period” was changed to “Mosquitoes can acquire the virus by feeding on a viraemic bird and can transmit it to other birds, animals or humans following an *extrinsic* incubation period”. The affiliations for Tjomme van Mastrigt were corrected from 4,8,9 to 3,8,9. These changes were published on 27 October 2020.

