# Supplementary material for: Detection of West Nile virus in a common whitethroat (Curruca communis) and Culex mosquitoes in the Netherlands, 2020
Source: Euro Surveill. 2020 Oct 8;25(40):2001704. doi: 10.2807/1560-7917.ES.2020.25.40.2001704 (PMC7545818; doi:10.2807/1560-7917.ES.2020.25.40.2001704)
Supplement: Supplement [file 20-01704_SIKKEMA_Supplementary_Table.pdf]

Supplementary table 1: West Nile Virus primer sequences and concentrations

This supplementary material is hosted by Eurosurveillance as supporting information alongside the article *First detection of West Nile Virus (WNV) in a Common Whitethroat (Curruca communis) and Culex mosquitoes in the Netherlands*, on behalf of the authors, who remain responsible for the accuracy and appropriateness of the content. The same standards for ethics, copyright, attributions and permissions as for the article apply. Supplements are not edited by Eurosurveillance and the journal is not responsible for the maintenance of any links or email addresses provided therein

**Mix 1**		**µl**	**Mix 2**		**µl**
WNVUS1_1_LEFT	GCCTGTGTGARCTGACAAACTTAG	10	WNVUS1_2_RIGHT_2	CGGGCTGTCAATATGCTAAAACGC	10
WNVUS1_1_RIGHT	CTTTTCTTTTGTTTTGRGCTCCG	10	WNVUS1_2_RIGHT	GTGCACCAGCAGTCAATGTCTTC	10
WNVUS1_3_LEFT	AGTTACCCTCTCTAACTTCCAAG	10	WNVUS1_2_LEFT	GTGCACCAACAGTCGATGTCTTC	10
WNVUS1_3_LEFT_2	GTGACCCTCTCCAACTTCCAGG	10	WNVUS1_4_LEFT	GGATGCTAGGAAGCAACACAATGC	10
WNVUS1_3_RIGHT	CARGAAGTCTCTGTTRCTCATTCC	10	WNVUS1_4_RIGHT_2	GATGCTTGGRAGCAACACCATG	10
WNVUS1_5_LEFT_2	GTGTCCAACCATGGGTGAAGCC	10	WNVUS1_4_RIGHT	TGCTYCCCTTTCCAAACAGTCC	10
WNVUS1_5_LEFT	GCCCGACCATGGGAGAAGCT	10	WNVUS1_4_LEFT_2	GCTTCCTTTGCCAAATAGTCCGC	10
WNVUS1_5_RIGHT_2	GTGGCATGAGGTTCTTCAAACTCC	10	WNVUS1_6_LEFT	GACTGTGARCCACGGTCAGG	10
WNVUS1_5_RIGHT	GGCGTGTGGTTCCTCAAACTCC	10	WNVUS1_6_RIGHT_2	CCGGTGTATTGCAGTTCCAACAC	10
WNVUS1_7_LEFT_2	TCTGAAGTGTAGGGTGAAGATGGAG	10	WNVUS1_6_RIGHT	GCAATTCCAACACCACAGTGCC	10
WNVUS1_7_LEFT	GTCATTTGAAGTGTAGAGTGAAGATGG	10	WNVUS1_8_LEFT_2	GTGAATCCATTTGTGTCTGTGGCC	10
WNVUS1_7_RIGHT	GAGGTGAAMACCCCTCCAACTG	10	WNVUS1_8_LEFT	GTCAACCCTTTTGTTTCAGTGGCC	10
WNVUS1_9_LEFT	GTGGATGGGMATCAATGCYCGT	10	WNVUS1_8_RIGHT_2	GATCCATCCAGGCTTCCACATC	10
WNVUS1_9_RIGHT_2	CTCTTGCCCCAAGCCTTCCAAC	10	WNVUS1_8_RIGHT	GGTCCATCCAAGCCTCCACATC	10
WNVUS1_9_RIGHT	CTTTCCCCAGGCCTTCCAGC	10	WNVUS1_10_LEFT_2	AGACTCGAGCACCAAATGTGGG	10
WNVUS1_11_LEFT	CACAACKGAATGYGACTCGAAGAT	10	WNVUS1_10_LEFT	CCAGACTGGAGCATCAAATGTGG	10

WNVUS1_11_RIGHT	ACGGTGTCCGCAGCTCTCAC	10	WNVUS1_10_RIGHT	GAACYGCCCTYTCAAGCTTCC	10
WNVUS1_11_RIGHT_2	CACGGTGTCCGCAACTRTCAC	10	WNVUS1_12_LEFT	GAAGTYAAATCATGYACSTGGCC	10
WNVUS1_13_LEFT_2	GGCACGACGAAAAGACCCTCGTGC	10	WNVUS1_12_RIGHT	CTTGCGAAGGACCTCCTGGG	10
WNVUS1_13_LEFT	GACATGATGAAAAGACCCTCGTGC	10	WNVUS1_14_LEFT_2	GTCCTAGTGTTTGGGGGTATTACG	10
WNVUS1_13_RIGHT_2	CTCTTGGTTGGTCCACCTTGC	10	WNVUS1_14_LEFT	CCTGGTGTTTGGGGGCATTAC	10
WNVUS1_13_RIGHT	CTCCTGGTTGGTCCATCTCGC	10	WNVUS1_14_RIGHT_2	GCAGATGAGGCAAGCYCCTTTC	10
WNVUS1_15_LEFT_2	CAAATGTGGTGGTGCCGCTGC	10	WNVUS1_14_RIGHT	CAAGCATARCAGACTTGCTCCTTTC	10
WNVUS1_15_LEFT	CGACATCAAACGTGGTTGTTCCG	10	WNVUS1_16_LEFT_2	CTGCAGTTGGACTCATGTTTGCC	10
WNVUS1_15_RIGHT_2	CYGTCCTCTCAATCCACATGTC	10	WNVUS1_16_LEFT	GCTGTCGGCYTRATGTTTGCCA	10
WNVUS1_15_RIGHT	CGCCGTTCTCTCAATCCACATATC	10	WNVUS1_16_RIGHT_2	GGTGATGGTGTGTCCCAAAGRAC	10
WNVUS1_17_LEFT_2	ATAAGTGCCTACACACCYTGGGC	10	WNVUS1_16_RIGHT	GAGGGAGTGTCCCACARCAC	10
WNVUS1_17_LEFT	GGAARATATGGATGCTCAGAATGG	10	WNVUS1_18_LEFT_2	CCACACACTATGGCACACCAC	10
WNVUS1_17_RIGHT_2	CCCCAATTTCTCCTTCTGGTGTC	10	WNVUS1_18_LEFT	GCAGGAGCRGGCGTGATG	10
WNVUS1_17_RIGHT	TTTGAACACCCCTGGTTTCGTC	10	WNVUS1_18_RIGHT_2	CTCARTCTTTTGTTGATGGCCTCC	10
WNVUS1_19_LEFT_2	CCATTGTGCAAGGAGAGAGAATGG	10	WNVUS1_18_RIGHT	GCCACAGATCATCAAAGAGGCC	10
WNVUS1_19_LEFT	CGGATTCGAACCTGAGATGCTG	10	WNVUS1_20_LEFT_2	GATGTCTCCACACAGAGTCCC	10
WNVUS1_19_RIGHT_2	CGATGCTCGCTGGATCCGTG	10	WNVUS1_20_LEFT	GATGTCTCCTCACAGGGTGCC	10
WNVUS1_19_RIGHT	CATGAATATTGCCGCCGCCTC	10	WNVUS1_20_RIGHT_2	GAAAGTCGTAYGAGACGGAGTAC	10
WNVUS1_21_LEFT_2	GGAAAGACCGTTTGGTTTGTTCC	10	WNVUS1_20_RIGHT	GGGTACTCTGTCTCATAGGACTTTC	10
WNVUS1_21_LEFT	GGGAAGACGGTTTGGTTTGTGC	10	WNVUS1_22_LEFT_2	GCTCAGCGGAGAGGACGC	10

WNVUS1_21_RIGHT_2	GAGTCGTCTTCATTCGTGTGCC	10	WNVUS1_22_LEFT	CGCCCAGAGACGTGGACG	10
WNVUS1_21_RIGHT	GTTGGAATCATCCTCATTTGTGTGC	10	WNVUS1_22_RIGHT_2	CTTTCTCTCACCCAACTTCGTG	10
WNVUS1_23_LEFT	CGGCTGGAGTGTCATACCACG	10	WNVUS1_22_RIGHT	GGCCTCAGAATCTTCCTTTCACC	10
WNVUS1_23_LEFT_2	CAGCAGGAATATCATACCATGACC	10	WNVUS1_24_LEFT_2	GATCACAAATCGGGCTCGTTGAG	10
WNVUS1_23_RIGHT_2	CTATTGTCTGAAGGGCGTCCGG	10	WNVUS1_24_LEFT	CGTTCTCAGATAGGGCTCATTGAG	10
WNVUS1_23_RIGHT	GAATACTCCCATGGTCATCACACTC	10	WNVUS1_24_RIGHT_2	CAACTCCCAGRGTCGTCTCTC	10
WNVUS1_25_LEFT_2	CAGGAACGAAAATAGCAGGCATGC	10	WNVUS1_24_RIGHT	CTCCTTGACCTCAATTCTTTGCCC	10
WNVUS1_25_LEFT	GGAACGAAGATCGCCGGAATG	10	WNVUS1_26_LEFT_2	GTGGACGTTGGTGTGTCAGCTC	10
WNVUS1_25_RIGHT_2	GCTTCCGCTTGCCAGCCTG	10	WNVUS1_26_LEFT	CTTCGTCGATGTTGGAGTGTCG	10
WNVUS1_25_RIGHT	GCTGAGCGCATTGCCTCAGC	10	WNVUS1_26_RIGHT_2	GTTGCATTCCACACTGAACTAGC	10
WNVUS1_27_LEFT_2	CAGTCATGCAGAAAAARGTTGGACAG	10	WNVUS1_26_RIGHT	CCAAACAGAGCTTGCTCCATTCTC	10
WNVUS1_27_LEFT	GATCTTGGTGTCTCTAGCTGCAG	10	WNVUS1_28_LEFT_2	GGGAAGTTTGGAAGGAGAGACTC	10
WNVUS1_27_RIGHT_2	CGAGATCCACAACCTTTCCCAC	10	WNVUS1_28_LEFT	GTACCGCAAAGAGGCCATCATC	10
WNVUS1_27_RIGHT	CATCCAAGGTCAATCACTTTTCCG	10	WNVUS1_28_RIGHT_2	CCAATGTCACAGAGCAGTGTGTC	10
WNVUS1_29_LEFT_2	CTGGCCATGAAGAGCCACAAC	10	WNVUS1_28_RIGHT	GARGACTCTCCGATGTCACAAAG	10
WNVUS1_29_LEFT	GTACAGGAAGTGAAAGGGTACACG	10	WNVUS1_30_LEFT_2	CCATGAGATGTACTGGGTGAGY	10
WNVUS1_29_RIGHT_2	GTTGACATCTTCCTCAAACTGGGG	10	WNVUS1_30_LEFT	GACTGGTCAGAAACCCACTCTC	10
WNVUS1_29_RIGHT	GCCCTGGTTCCACTTCCCAAG	10	WNVUS1_30_RIGHT_2	GAAGGGAGTAGTGTCAGTCATGG	10
WNVUS1_31_LEFT_2	GAATACAGCTCCACATGGCACC	10	WNVUS1_30_RIGHT	CACTCGTTGTTGACCGAAAGGAG	10
WNVUS1_31_LEFT	GAGAACCACCCATATAGAACCTGG	10	WNVUS1_32_LEFT_2	GGAAGAACGCCCGGGAAGC	10

WNVUS1_31_RIGHT	CTCTTTCCCATCATGTTGTARATGC	10	WNVUS1_32_LEFT	GAGGAGCGCCAGAGARGCAG	10
WNVUS1_33_LEFT_2	GGGTACATCTTGAAGGAAGTYGG	10	WNVUS1_32_RIGHT_2	CAGCAGTTCAAGAACCTTCGCTTC	10
WNVUS1_33_LEFT	GTTACATCCTGCGTGAAGTTGGC	10	WNVUS1_32_RIGHT	CCAAGTCAGCTCTCGTGATGCG	10
WNVUS1_33_RIGHT	CSCCATTCTCAAACAGCCAGG	10	WNVUS1_34_LEFT_2	GTGAAAGTGATGCGCCCGGC	10
WNVUS1_35_LEFT_2	GGTGGTATGACTGGCAGCAGG	10	WNVUS1_34_LEFT	GTCGTGAAAGTGATGAGGCCAG	10
WNVUS1_35_LEFT	GATGGTATGATTGGCAGCAGGTTC	10	WNVUS1_34_RIGHT_2	GAGAWATGCGAGCTCTGCCTAC	10
WNVUS1_35_RIGHT	GTCTTCCATCCAYTCATTCTCCTC	10	WNVUS1_34_RIGHT	CGCACATTCCATCCAGCCCC	10
WNVUS1_37_LEFT	GAGAAGTATGYGGATTACATGAGYTC	10	WNVUS1_36_LEFT_2	CGCAAAAGGAGAATGGATGACGAC	10
WNVUS1_37_RIGHT	GGTCTCCTCTAACCTCTAGTCC	10	WNVUS1_36_LEFT	CCATGCAGGAGGAGAGTGGATG	10
			WNVUS1_36_RIGHT	CGTCTACTCAACTTCCGGTGG	10
			WNVUS1_38_LEFT	CCCTCAGAACCGTCTCGGAAG	10
			WNVUS1_38_RIGHT	GCACTGTGCCGTGTGGCTG	10
